# An *ex vivo* RNA *trans*‐splicing strategy to correct human generalized severe epidermolysis bullosa simplex

**DOI:** 10.1111/bjd.17075

**Published:** 2018-10-07

**Authors:** P. Peking, J.S. Breitenbach, M. Ablinger, W.H. Muss, F.J. Poetschke, T. Kocher, U. Koller, S. Hainzl, S. Kitzmueller, J.W. Bauer, J. Reichelt, T. Lettner, V. Wally

**Affiliations:** ^1^ EB House Austria Research Program for Molecular Therapy of Genodermatoses Department of Dermatology University Hospital of the Paracelsus Medical University Salzburg Müllner Hauptstraße 48 5020 Salzburg Austria; ^2^ Cell Therapy Institute Spinal Cord Injury and Tissue Regeneration Center Salzburg (Sci‐TReCS) Paracelsus Medical University Salzburg Austria; ^3^ Institute of Pathology University Hospital of the Paracelsus Medical University Salzburg Salzburg Austria; ^4^ Department of Dermatology University Hospital of the Paracelsus Medical University Salzburg Salzburg Austria

## Abstract

**Background:**

Generalized severe epidermolysis bullosa simplex (EBS‐gen sev) is a genetic blistering skin disease in which autosomal dominant mutations in either the keratin *KRT5* or *KRT14* genes lead to impaired function of the intermediate filament cytoskeleton in the basal epidermis. Here we present an *ex vivo* RNA *trans*‐splicing‐based therapeutic approach to correct the phenotype.

**Objectives:**

To correct a mutation within exon 1 of the *KRT14* gene, using a 5′‐*trans*‐splicing approach, where any mutation within the first seven exons could be replaced by a single therapeutic molecule.

**Methods:**

A therapeutic RNA *trans*‐splicing molecule containing wild‐type exons 1–7 was stably transduced into an EBS patient‐derived keratinocyte line. *Trans*‐splicing was confirmed via reverse‐transcriptase polymerase chain reaction, Western blotting and immunofluorescence microscopy. Skin equivalents generated from corrected keratinocytes were grafted onto nude mice and analysed about 8 weeks post‐transplantation for regular epidermal stratification, *trans*‐splicing‐induced green fluorescent protein expression and blistering.

**Results:**

Transplanted skin equivalents generated from *trans*‐splicing‐corrected patient keratinocytes showed a stable and blister‐free epidermis. *KRT14* correction disrupted EBS‐gen sev‐associated proinflammatory signalling, as shown at the mRNA and protein levels. Disruption of the pathogenic feedback loop in addition to overall downregulation of *KRT14* expression highlighted the effect of *KRT14* correction on the EBS pathomechanism.

**Conclusions:**

Our data demonstrate that *trans*‐splicing‐mediated mRNA therapy is an effective method for the correction of dominantly inherited *KRT14* mutations at the transcriptional level. This results in the rescue of the EBS‐gen sev phenotype and stabilization of the epidermis in a xenograft mouse model.

## Abstract 2

**What's already known about this topic?**

- RTM163, described in this study, was previously used in a transient *in vitro* transfection system, where the ability to correct *KRT14* at the mRNA level was demonstrated.

**What does this study add?**

- In this study, we stably transduced RTM163 in a second patient‐derived keratinocyte line.
- Successful *trans*‐splicing was confirmed in this cell line.
- The expression of disease‐related marker genes, which are characteristically deregulated in epidermolysis bullosa simplex, were analysed.
- For the first time this study showed that RNA *trans*‐splicing molecule‐transduced patient keratinocytes can differentiate into a phenotypically normal and blister‐free epidermis in a xenograft mouse model.

**What is the translational message?**

- This study shows the feasibility of using spliceosome‐mediated RNA *trans*‐splicing to generate a stable and blister‐free epidermis *in vivo*.
- Combined with pre‐existing *ex vivo* gene therapeutic methods, this might be a valid option for future treatments of dominantly inherited genodermatoses.

**Linked Comment:** Bremer and van den Akker. *Br J Dermatol* 2019; **180**:17–19.

Devising efficient therapies for dominantly inherited diseases such as epidermolysis bullosa simplex (EBS) remains a challenge. Detailed knowledge of the mutation‐associated pathomechanism, as in the case of EBS, can improve therapeutic success by providing a molecular readout of disease markers. Targeting epidermolysis bullosa (EB) mutations at the genomic level would provide permanent correction.^1^ However, an alternative approach, based on replacing defined segments of the mutant mRNA via therapeutic RNA molecules, would be applicable to a broad patient cohort. Furthermore, the most advanced clinical gene replacement therapy cannot overcome the effect of a dominantly interfering allele,^2^, ^3^, ^4^ even though it has been successfully applied in numerous cases of recessively inherited gene defects such as the EB‐associated gene *LAMB3*. This underlines that there is no current therapy without potential drawbacks, but only a set of therapeutic options that can be applied according to the specific setting and genetic disease.

Several studies using spliceosome‐mediated mRNA *trans*‐splicing (SMaRT) have proved the feasibility of this approach to correct dominant and recessive mutations at the mRNA level, by replacing sequences of up to 4·5 kb.^5^, ^6^ The basis of SMaRT is the engineering of an RNA *trans*‐splicing molecule (RTM). This provides (i) the coding region to be replaced, either a 5′‐, 3′‐ or central gene portion; (ii) functional, and in particular strong splice sites that enhance *trans*‐ over *cis*‐splicing; and (iii) a binding domain that hybridizes with a distinct target mRNA. This is required for the two pre‐mRNAs to be brought into apposition, facilitating *trans*‐splicing.^7^ The functionality of this approach has previously been shown in cystic fibrosis,^8^, ^9^ spinal muscular atrophy,^10^ Duchenne muscular dystrophy,^11^ DNA protein kinase deficiency,^12^ Huntington disease,^13^ X‐linked immunodeficiency^14^ and many more conditions. Furthermore, we have previously demonstrated successful correction of mRNAs containing mutations associated with numerous EB subtypes.^15^, ^16^, ^17^, ^18^, ^19^, ^20^

Among the heterogeneous group of genodermatoses, EB is characterized by blistering and erosions of the skin and mucous membranes as a result of a defect in anchoring between the epidermis and dermis. To correct a dominantly inherited hotspot mutation (c.373C>T; p.R125H) in the keratin 14 gene (*KRT14*), which causes the generalized severe EBS subtype (EBS‐gen sev), we devised a 5′‐*trans*‐splicing strategy.^21^ In basal keratinocytes, which comprise the majority of cells within the basal epidermal layer, *KRT14* encodes the intermediate filament protein keratin 14. EBS‐gen sev is considered one of the more severe *KRT14*‐associated EBS subtypes. This is due to a constitutively activated proinflammatory stress pathway, triggered via pathological accumulation of keratin 14‐containing aggregates in the keratinocyte cytoplasm.

Keratin 14 heteropolymerizes with keratin 5 to build the keratin intermediate filament (KIF) network. This network spans the cytoplasm, attaching to desmosomes and hemidesmosomes. These plaque‐like structures connect keratinocytes to neighbouring cells and the basement membrane, respectively. Dominant point mutations in either of these two keratin genes most frequently affect the ends of the central rod domains of keratins 14 and 5, which are responsible for heterodimerization, leading to collapse of the KIF network upon minor mechanical trauma. This also results in characteristic KIF aggregates, which accumulate in the periphery of the cytoplasm. Apparently, as the cells try to dispose of these aggregates, the proinflammatory cytokine interleukin (IL)‐1β is secreted and activated at increased levels, as also described for Huntington disease.^22^, ^23^

Along with creating an inflammatory milieu within the skin, IL‐1β also triggers the c‐Jun *N*‐terminal kinase (JNK)‐mediated stress pathway in an autocrine manner. This, in turn, activates the transcription of AP‐1‐responsive genes such as matrix metalloproteinase 1 (*MMP1*), kallikrein 7 (*KLK7*), rho guanine nucleotide exchange factors (ARHGEF family) and others, and also *KRT14* and *IL1B* themselves.^24^ The resultant positive feedback loop and overexpression of keratin 14 are ultimately the reason for the severe phenotype of EBS‐gen sev. Disruption of this feedback loop in EBS keratinocytes via inhibition of IL‐1β leads to stabilization of the KIF network, restoring its resistance to mechanical friction.^23^

Currently, treatment of EBS‐gen sev is confined to the treatment of symptoms and standard wound care.^25^ First clinical trials have been published, in which small molecules were used to alleviate secondary manifestations of the disease, in an untargeted manner. We recently demonstrated the impact of the small molecule diacerein on the IL‐1β‐induced JNK stress pathway. This was shown *in vitro* and resulted in a clinically meaningful reduction of blisters in a pilot and a phase II/III clinical trial.^26^ The use of diacerein, or other substances targeting the JNK stress pathway (e.g. anakinra, rilonacept), will provide only a transient effect on blister reduction. However, their potentially short‐term availability currently renders such approaches an attractive option for increasing patients’ quality of life. Especially for causal therapies, which aim to correct genetic mutations, several hurdles remain.^26^, ^27^

In this study we demonstrate the feasibility of SMaRT to reduce keratin 14 overexpression and to phenotypically correct patient keratinocytes in a xenograft mouse model. Supporting previous findings of the pathomechanism, our data show that a reduction in the expression of the mutated allele in comparison with the wild‐type allele upon *trans*‐splicing‐mediated correction results in downregulation of IL‐1β‐mediated JNK‐signalling, indicating the potential high impact of this treatment in EBS.

## Materials and methods

### Cloning of the RV_RTM163_GFP construct

The original construct, RTMe163, was cloned into the retroviral vector pLXSN for stable transduction of patient keratinocytes.^19^ The RTM, which contains *KRT14* exons 1–7, as well as a 170‐bp binding domain complementary to *KRT14* intron 7, was cloned in fusion with (RV_RTM163_GFP) or without (RV_RTM163) a 5′‐full‐length green fluorescent protein (GFP). Additionally, a *KRT14* promoter was cloned upstream of the RTM‐expression cassette to provide tissue‐specific expression of the RTM.

### Cell lines

The keratinocyte lines used in this study are the previously published EBS‐gen sev cell line EBDM‐1 (*KRT14*, c.373C>T, p.R125H) and the human wild‐type keratinocyte lines hKc and NEB‐1.^24^, ^28^ Cells were cultured in a humidified incubator at 37 °C and 5% CO_2_ using EpiLife medium (Thermo Fisher Scientific Inc., Waltham, MA, U.S.A.) supplemented with Human Keratinocyte Growth Supplement (Cascade Biologics, Bromborough, U.K.) and 10% penicillin–streptomycin (Biochrom AG, Berlin, Germany). The medium was replaced every second day and cells were passaged at a confluence of 80%.

### Transplantation of organotypic three‐dimensional skin equivalents onto nude mice

Animal trial permission was given by the local authorities according to Austrian legislation (§26 TVG 2012; animal trial number 20901‐TVG/58/22‐2014).

Transplantation of three‐dimensional skin equivalents was performed as previously described.^29^ Briefly, female, immunodeficient nude mice (Crl:CD1‐Foxn1nu, strain code 086; Charles River Laboratories, Rhön‐Grabfeld, Germany) were delivered at age 6–8 weeks and kept in the specific pathogen‐free animal facility for 2 weeks. Skin equivalents were transplanted onto the dorsal region of the mice. The excised mouse skin was devitalized using liquid nitrogen and used as a cover for the transplant, coming off naturally after about 10 days. Grafted skin equivalents were left to differentiate for 8 weeks before harvesting.

Additional methods are described in Appendix S1 (see Supporting Information).

## Results

### RTMe163‐transduced EBDM‐1 keratinocytes show specific *trans*‐splicing

EBDM‐1 keratinocytes were used in order to confirm that this construct is functional in another EBS‐gen sev cell line upon retroviral transduction (RV_RTM163_GFP). EBDM‐1 keratinocytes, harbouring a p.R125H mutation within the keratin 14 helix initiation motif, were transduced with RV_RTM163_GFP, and GFP‐expressing EBDM‐1 keratinocytes were enriched by fluorescence‐activated cell sorting to obtain a homogeneously transduced cell population (Fig. S1; see Supporting Information).

**Figure 1:**
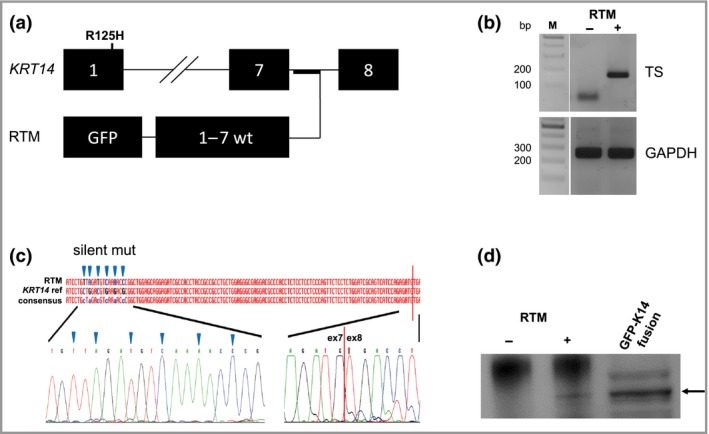
*Trans*‐splicing in RNA *trans*‐splicing molecule (RTM)‐transduced EBDM‐1 keratinocytes. (a) RV_RTM163_GFP hybridizes to *KRT14* intron 7 to facilitate *trans*‐splicing, resulting in replacement of endogenous *KRT14* exons 1–7 with an RTM‐provided wild‐type (wt) sequence. (b) Reverse‐transcriptase polymerase chain reaction revealed a 187‐nucleotide *trans*‐splicing (TS)‐specific band in RTM‐transduced (RTM+) patient keratinocytes. Negative control, untransduced patient keratinocytes (RTM–); reference gene, *GAPDH*. (c) Sequence analysis confirms successful *trans*‐splicing, showing the exon 7–exon 8 junction and inclusion of silent mutations (triangles) in the RTM‐derived *KRT14* coding region. (d) Western blot analysis revealed a band of the same molecular weight as a control green fluorescent protein (GFP)–keratin 14 (K14) fusion protein in RTM+ keratinocytes.

Five silent mutations, included in exon 6 of the RTM, enable discrimination between *cis‐* and *trans*‐spliced mRNA (Fig. 1a). *Trans*‐splicing was specifically detected using reverse‐transcriptase polymerase chain reaction (PCR) and forward primers specific for (i) the endogenous *KRT14* exon 6/7 junction to amplify total *KRT14* mRNA and (ii) the introduced silent mutations within exon 6 of the RTM coding region, both together with an exon 8 reverse primer. *Trans*‐splicing accuracy to endogenous *KRT14* exon 8 was confirmed by sequence analysis of the 157‐nucleotide PCR product (Fig. 1b, c). At the protein level, a GFP–keratin 14 fusion product was detected in Western blot analysis upon immunoprecipitation of the chimera using a GFP antibody (Fig. 1d).

### Phenotypic correction of EBDM‐1_RTM163_GFP‐derived skin grafts

**Figure 2:**
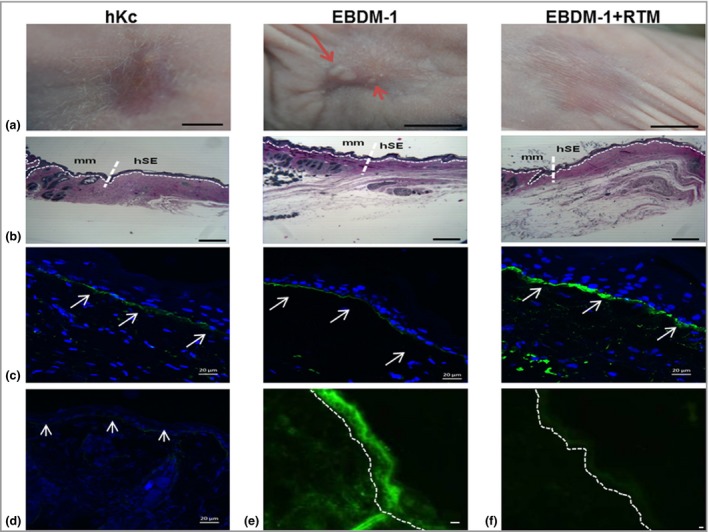
Phenotypic and molecular characterization of skin equivalents. (a) Spontaneous blistering occurred only in uncorrected keratinocyte‐derived skin equivalents derived from a patient with epidermolysis bullosa simplex (red arrows). Scale bars = 1 cm. hKc, human keratinocyte. (b) Azure–methylene blue–basic fuchsin‐stained resin sections of explants show the junction between murine (mm, left) and human (hSE, right) tissue. Scale bars = 100 μm. (c) C7 expression at the dermoepidermal junction (DEJ) is visible on explanted skin sections and respective controls (white arrows), but not in mouse tissue (d). Scale bars = 20 μm. (e) Skin sections derived from EBDM‐1+RTM (RNA *trans*‐splicing molecule) transplants show *trans*‐splicing‐derived green fluorescence in the human skin equivalent part, barely and unspecifically detectable in murine tissue (f). Scale bars = 20 μm. Dotted lines show the DEJ.

To investigate the capability of RTM‐transduced EBDM‐1 keratinocytes to differentiate and stratify into phenotypically corrected skin *in vivo*, we followed an *ex vivo* gene therapeutic approach, in which three‐dimensional skin equivalents were transplanted onto the back of nude mice. Skin equivalents differentiated fully and, at the time of excision, phenotypically resembled normal human skin. Three transplants derived from EBDM‐1 keratinocytes (from a total of 11 transplants) demonstrated spontaneous blistering, whereas EBDM‐1+RV_RTM163_GFP‐derived transplants (*n* = 7) revealed a phenotype like the control grafts (hKc; human wild‐type keratinocyte derived, *n* = 11), showing no signs of any blister formation (Fig. 2a).

Morphological examination of semithin sections of excised grafted areas showed a clear boundary between the recipient mouse and the transplanted human skin tissue. Whereas the mouse tissue showed abundant skin appendages, the human grafts were devoid of any epidermal appendages. A normal‐looking epidermis, consisting of at least three cell layers and a stratum corneum, was present in all grafts (Fig. 2b). Ultrastructural analysis of the transplanted skin equivalents revealed keratin filament bundle formation in transplants generated from RTM‐expressing patient keratinocytes, whereas in noncorrected transplants more keratin aggregates were noted (Fig. S2; see Supporting Information).

**Figure 3:**
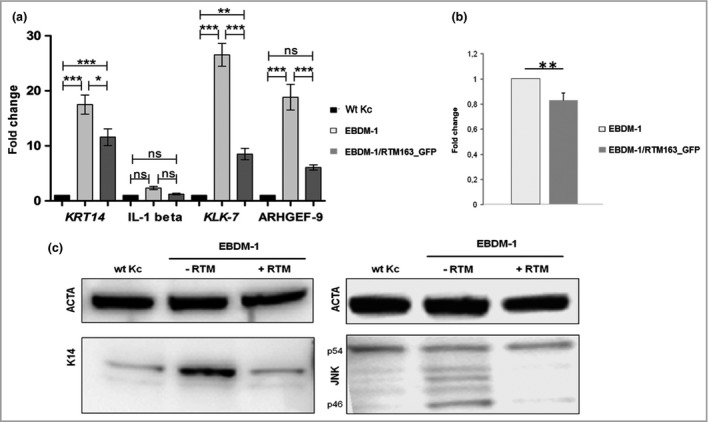
Impact of *trans*‐splicing on molecular players involved in the pathomechanism of generalized severe epidermolysis bullosa simplex. (a) Semiquantitative reverse‐transcriptase polymerase chain reaction: downregulation of *KRT14*,*IL1B*,*KLK7* and *ARHGEF9* mRNA in EBDM‐1/RV‐RTM163_GFP compared with untreated EBDM‐1 keratinocytes. Data represent the means and SDs of four (*IL1B*,*KLK7*) or five (*KRT14*,*ARHGEF9*) independent experiments, one‐way anova: ***P < 0·001; **P < 0·01; *P < 0·05; ns, not significant. Wt Kc, wild‐type keratinocyte. (b) Luciferase assay. Upon *trans*‐splicing, the luciferase signal decreased significantly in EBDM‐1/RV_RTM163 keratinocytes compared with EBDM‐1 cells (*n* = 5). Statistics: two‐sided independent Student's *t*‐test; **P < 0·01. (c) Western blot analysis: RNA *trans*‐splicing molecule (RTM)‐corrected EBDM‐1 cells show similar levels of keratin 14 (K14) expression and c‐Jun *N*‐terminal kinase (JNK) phosphorylation (especially the p46 variant) compared with normal. ACTA, α‐actin control.

To verify the human origin of the skin equivalents, we isolated genomic DNA from punch biopsies taken from the centre of the explants. This was subjected to PCR amplification and sequencing to confirm the presence of the RTM in the transplanted keratinocytes; no band was detectable in mouse genomic DNA (Fig. S3; see Supporting Information). In addition, a human type VII collagen‐specific antibody demonstrated type VII collagen deposition at the basement membrane zone of the skin equivalent, but not within mouse skin (Fig. 3c, d). Finally, GFP expression was visible in the human graft region only, indicating expression of a *trans*‐splicing‐derived GFP–keratin 14 fusion protein (Fig. 2e, f). In all transplanted skin equivalents, keratin 14 and 5 expression was noted in the basal keratinocyte layers, whereas keratin 1 was expressed in the suprabasal layers, confirming correct differentiation and stratification. Staining for Ki67 showed that approximately 10–15% of the basal keratinocytes were proliferating (Fig. S4; see Supporting Information).

### Impact of *KRT14 trans*‐splicing correction on key players in the pathomechanism of generalized severe epidermolysis bullosa simplex

In a previous study, we showed that the IL‐1β‐mediated JNK stress pathway is constitutively activated in EBS‐gen sev keratinocytes as a consequence of cytoplasmic keratin 14 protein aggregation.^24^ We further showed that this results in increased transcriptional levels of AP‐1‐responsive genes, among them *KRT14* and *IL1B*, in a positive feedback loop, which exacerbates the phenotype due to accumulation of keratin 14 (wild‐type and mutated) in the cytoplasm. We also demonstrated that downregulation of *KRT14* expression ameliorates EBS‐gen sev hallmarks *in vitro* and *in vivo*.^24^, ^26^, ^27^

In order to examine the effect of *trans*‐splicing‐mediated *KRT14* correction on the JNK stress pathway, we assayed *KRT14* promoter‐driven luciferase expression, based on the hypothesis that a decrease in mutated keratin 14 and a concomitant reduction of cytoplasmic aggregates would reduce *KRT14* promoter activity. Indeed, luciferase activity was significantly decreased in RTM‐transduced EBDM‐1 keratinocytes in comparison with nontransduced EBDM‐1 cells (Fig. 3a). Additionally, we found reduced expression levels of the JNK downstream targets *KRT14*,* IL1B*,* KLK7* and *ARHGEF9* by semiquantitative reverse‐transcriptase PCR (Fig. 3b).

Western blot analysis confirmed a significant reduction of overall keratin 14 expression in RTM‐transduced EBDM‐1 vs. nontransduced cells, approximating the level detected in wild‐type keratinocytes. Furthermore, decreased phosphorylation of JNK, especially of the p46 variant, which is reported to be the one most responsive to extracellular stimuli like IL‐1β, was clearly detectable in RTM‐transduced EBDM‐1 cells (Fig. 3c).^30^ Overall JNK expression was unchanged (data not shown).

## Discussion

Treatment of medically intractable hereditary diseases requires replacement or correction of the affected gene or RNA in order to facilitate expression of a functional wild‐type protein. Whereas cDNA therapy was previously shown to be an option for many recessively inherited genetic diseases in clinical trials, therapy of dominant negative mutations requires different approaches, which will either repair or knock down the disease‐causing allele or gene product.^1^, ^3^, ^4^, ^31^, ^32^ Such genome editing approaches might involve CRISPR (clustered regularly interspaced short palindromic repeats) or TALEN (transcription activator‐like effector nuclease) technologies. These would either alter sequences at the genetic level, thereby activating premature termination codons to knock down a specific mutated allele, or induce homology‐directed repair if an appropriate template is provided.^33^, ^34^

Spliceosome‐mediated RNA *trans*‐splicing has the advantage of producing concomitant knock‐down of the mutated transcript while increasing the number of wild‐type transcripts in a single reaction, without the necessity for allele specificity.^7^ Additionally, a single therapeutic molecule can correct multiple mutational sites within a transcript. Therefore, considering that the mutational hotspots for EBS‐gen sev are located in exons 1 and 6 of the *KRT14* gene, one therapeutic molecule is employable for the vast majority of patients harbouring mutations in *KRT14*. In practical terms, this could reduce the costs of developing the technology.

Furthermore, RNA *trans*‐splicing lowers certain risks compared with genome editing. This is a result of the intervention occurring during the naturally occurring splicing reaction and an absence of externally induced DNA or RNA breaks. However, for permanent RNA *trans*‐splicing correction of the gene defect, stable viral integration is required, accompanied by risks such as insertional mutagenesis and generation of DNA rearrangements during the viral packaging.^35^, ^36^ The well‐established *ex vivo* gene therapy, whereby autologous stem cells have been retrovirally transduced with a full‐length cDNA copy of the gene of interest prior to transplantation,^2^, ^3^, ^4^ enables monitoring of potential side‐effects prior to transplantation. Such a strategy might be employed in a *trans*‐splicing approach for the permanent treatment of specific sites of the body such as the feet, which are particularly prone to blistering in patients with EBS‐gen sev.

A common challenge for most gene or RNA therapeutic approaches is targeted delivery of the nucleic acid to the appropriate cells. In the case of EBS, cellular targets are epidermal stem cells located in the basal layer of the epidermis. In our study, we separated retrovirally RTM+GFP‐transduced keratinocytes (~15%) from the bulk population by fluorescence‐activated cell sorting prior to transplantation, producing a homogeneous RTM‐transduced cell population. For potential clinical applications, the use of clinical‐grade vectors lacking any reporter molecules for cell preselection is necessary. Thus, high transduction rates are crucial, requiring optimal selection of the vector. To infect keratinocytes with a low cell division rate, the use of a lentiviral vector system might be an option, due to the ability of this system to infect dividing and nondividing cells.^37^ Additionally, we recently showed that isolation and expansion of RTM‐transduced keratinocyte single‐cell clones resulted in a homogeneous cell population expressing the desired transgene.^18^

With regard to applying SMaRT as a local *in vivo* therapy approach, we recently showed that nonviral gene‐gun‐mediated delivery of an RTM engineered to reprogram murine *Col7a1* resulted in the expression of the *trans*‐splicing protein product at the dermoepidermal junction in a mouse model. The gold‐particle carriers for the RTM were localized in the epidermis. This suggested that targeting of dividing keratinocytes within the basal epidermal layer was very likely achieved, positioning this technology as an option for *KRT14 trans*‐splicing in *in vivo* models.^17^ However, the corrective effect is reversible when using nonviral vectors for *in vivo* skin delivery, requiring a continuous repetition of the treatment.

Recently, liposomes were shown to pass through the stratum corneum and deliver short RNAs into epidermal cells.^38^ Presently, these have begun to be used in clinics and initial products have been approved. Although drawbacks and limitations of liposome‐based delivery have also been reported, they are currently considered a successful drug delivery system with a high potential for expanding the spectrum of clinical applications.^39^ Liposomes could be used to facilitate repeated applications of SMaRT correction, such as at cutaneous sites that experience high levels of stress. Despite the need for repeated applications due to the transient effects of the therapy in this setting, alterations at the genomic level can be excluded. Therefore, discontinuation of the therapy in the case of unexpected side‐effects is rapid and simple, increasing its safety. However, effective and precise delivery of RNA molecules into the skin, and in particular into keratinocyte stem cells, remains a major hurdle.

The inclusion of a tissue‐specific *KRT14* promoter, driving the expression of the RTM, increases specificity and decreases the risk of side‐effects upon potential transdermal delivery and uptake of the RTM by other cell types. Overexpression of *KRT14* in keratinocytes leads to aggregation of the KIF, demonstrated both *in vitro* 40 and *in vivo* in an inducible mouse model for EBS.^41^ Because the SMaRT approach relies on the endogenous expression pattern – temporal and spatial – *KRT14* overexpression is circumvented.

In summary, our data demonstrate that *trans*‐splicing‐mediated mRNA therapy is an effective method for the correction of dominantly inherited *KRT14* mutations. For the first time we have shown the impact of *KRT14 trans*‐splicing correction on the EBS‐gen sev‐associated, constitutively activated IL‐1β‐mediated stress pathway, as well as the reversion of the EBS‐gen sev phenotype by producing a stable epidermis in a xenograft mouse model.

## Supporting information


**Appendix S1** Supplementary materials and methods.
**Fig S1.** Sorting of RNA *trans*‐splicing molecule‐expressing keratinocytes.
**Fig S2.** Ultrastructure of the dermoepidermal junction zone of the xenografts.
**Fig S3.** Characterization of skin equivalents.
**Fig S4.** Differentiation, stratification and proliferation of transplanted skin equivalents.
