# Supplementary material for: An ex vivo RNA trans‐splicing strategy to correct human generalized severe epidermolysis bullosa simplex
Source: Br J Dermatol. 2018 Oct 7;180(1):141–8. doi: 10.1111/bjd.17075 (PMC6334280; doi:10.1111/bjd.17075)
Supplement: Supplementary file 1 — Appendix S1 Supplementary materials and methods. Fig S1. Sorting of RNA trans‐splicing molecule‐expressing keratinocytes. Fig S2. Ultrastructure of the dermoepidermal junction zone of the xenografts. Fig S3. Characterization of skin equivalents. Fig S4. Differentiation, stratification and proliferation of transplanted skin equivalents. [file BJD-180-141-s001.docx]

**Supporting Information:**

*Supporting Figure 1*

**
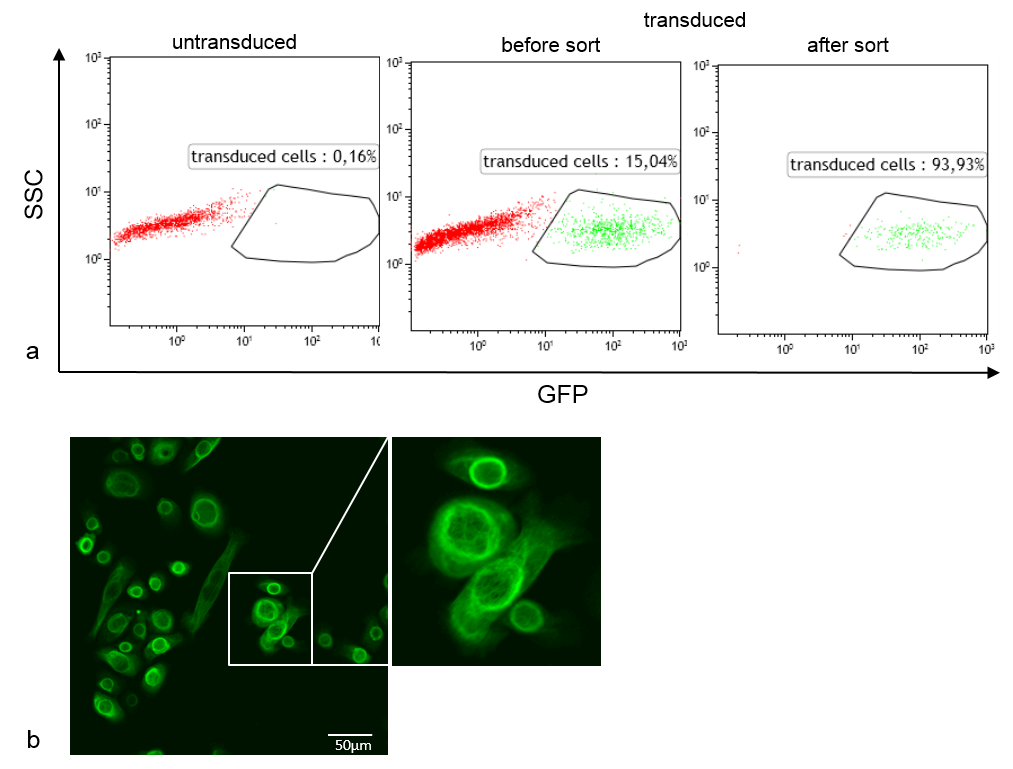
**

**Supporting Figure 1:** **Sorting of RTM-expressing keratinocytes.** a) In order to obtain a homogenous population of RTM-transduced keratinocytes, GFP-positive cells were sorted for further experiments. b) GFP localization in sorted RTM-expressing keratinocytes.

*Supporting Figure 2*


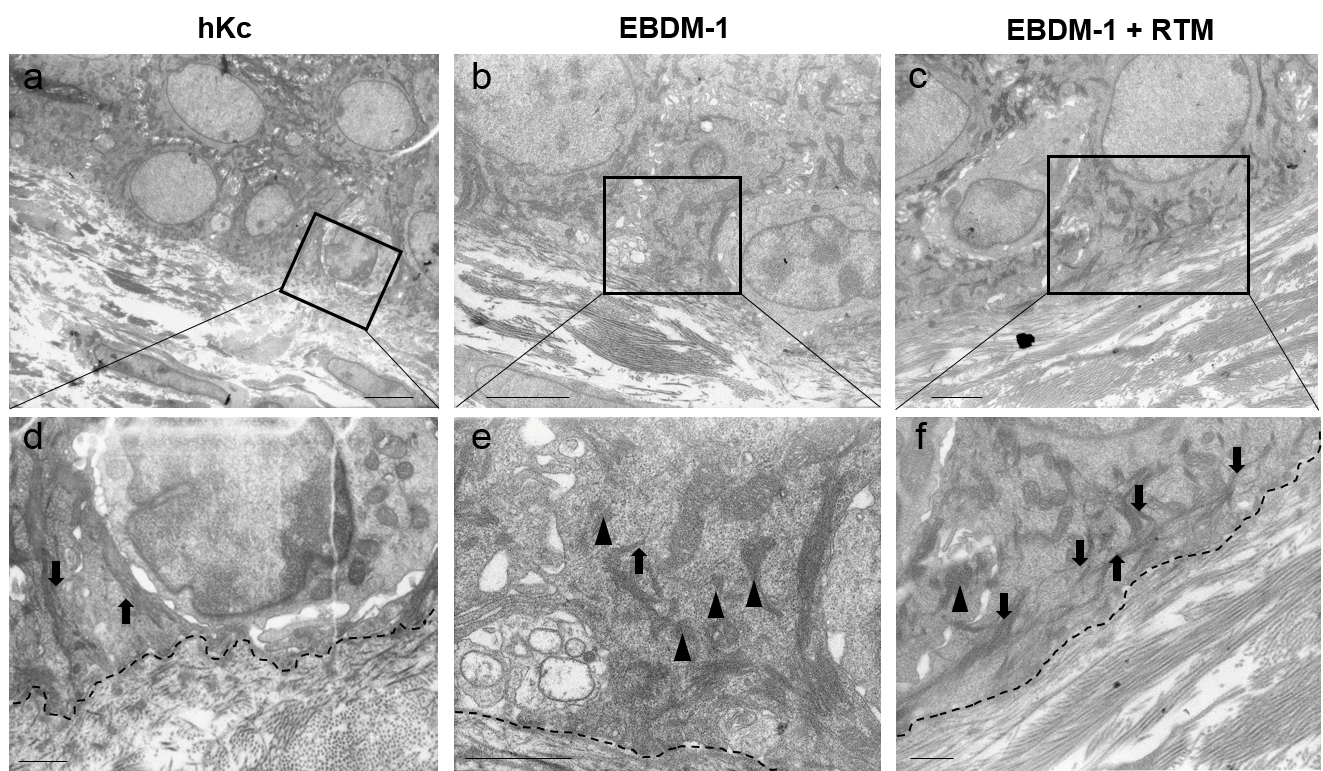


**Supporting Figure 2: Ultrastructure of the dermo-epidermal junction zone of the xenografts**. a-c) Overviews and magnifications (d-f) of the lower epidermis and the adjacent dermis of xenografts generated from control (hKc), unmodified EBDM-1 and RTM-transduced EBDM-1 cells are shown. The dashed lines indicate the basement membrane (d-f). Keratin filament bundles (arrows) are predominantly visible in hKc (a,d) and RTM transduced (EBDM-1 + RTM, B,E) SEs. The non-RTM-transduced SEs (EBDM-1, show several keratin aggregates (arrowheads). Scale bars: upper panel 10 µm, lower panel 2 µm.

*Supporting Figure 3*


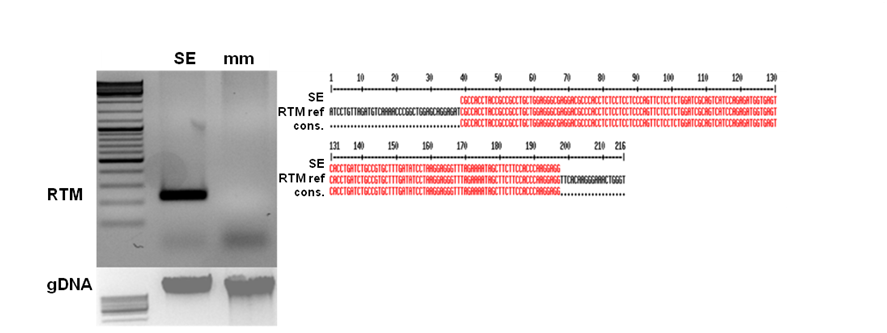


**Supporting Figure 3: Characterization of skin equivalents.** RTM-specific PCR was done on genomic DNA (gDNA) isolated from a SE derived from EBDM-1_RTM_GFP keratinocytes. A distinct band was amplified from SE-derived gDNA, but not from mouse skin (mm). As a control, gDNA is shown for both sample types. Sequence alignment with the RTM as a reference is shown.

*Supporting Figure 4*

*
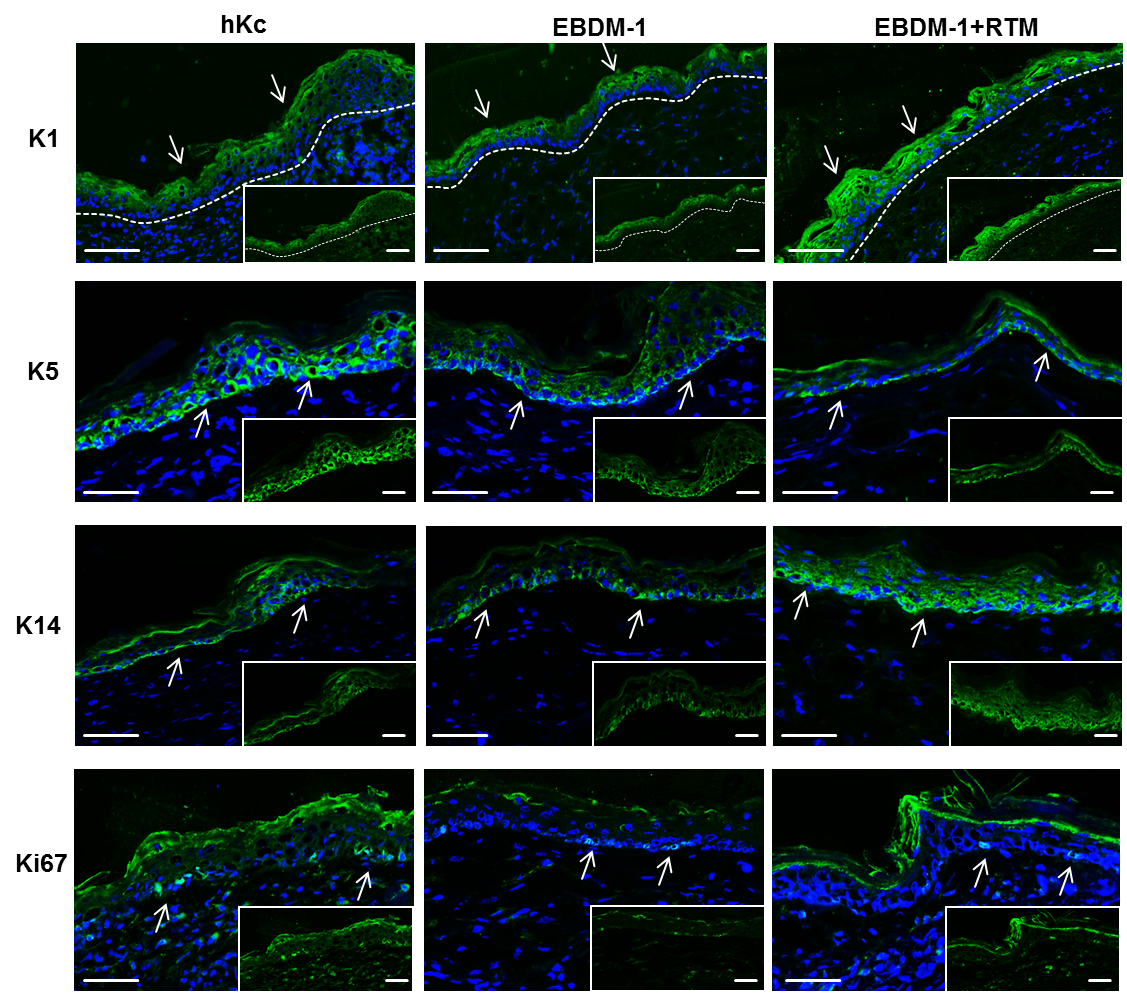
*

**Supporting Figure 4: Differentiation, stratification and proliferation of transplanted SEs.**  Immunofluorescence staining using antibodies specifically detecting K5 and K14, expressed in the basal keratinocyte layer and K1, expressed in the suprabasal layer is shown for all transplanted SEs (green). Proliferative cells within the SEs are positive for Ki67 staining (green). Cell nuclei are stained with DAPI (blue). Scale bars=50 µm. Dotted lines: dermo-epidermal junction.

**Material and Methods:**

*Viral integration*

Viral particle production: 293T human embryonic kidney (HEK) cell-based phoenix retrovirus producer cells were seeded in DMEM Hyclone medium supplemented with 10% inactivated FCII (Hyclone, VWR, Darmstadt, Germany), 2 mM L-glutamine and 1 mM Na-pyruvate. Cells were transfected at a confluence of 60% with 8 µg RV_RTM163_GFP plasmid using jetPEI reagent (Polyplus, Illkirch, France). For immortalizing primary keratinocytes, pLXSN-E6/E7-G418 vector (TaKaRa Bio Inc., Shiga, Japan) was used. Transfected cells were incubated at 32°C / 5% CO_2_ in a humidified incubator and the medium was changed after 24 hours. Viral supernatant was harvested every 8 hours and kept at 4°C until infection.

Viral transduction: For viral transduction, supernatants were filtered through a 0.22-µm filter and 5 µg/ml polybrene (Sigma, Vienna, Austria) were added. Supernatants were pipetted onto 0.5 x 10^6^ target cells at 50% confluence. Cells were centrifuged for 1.5 hours at 32°C and 600 x g and incubated at 32°C for 24 hours. Cells were sorted for GFP expression 2 weeks after transduction.

*Semi-quantitative real-time PCR*

Total RNA was isolated from cultured keratinocytes using an RNeasy Mini Kit (Qiagen, Hilden, Germany). Plasmid and genomic DNA contamination were eliminated by treatment with DNase I (Sigma, Vienna, Austria). cDNA was synthesized from 100 ng total RNA using an iScript cDNA Synthesis Kit (Bio-Rad, Vienna, Austria). Semi-quantitative real-time PCR (SQRT-PCR) was performed using the BIO-RAD CFX96™ Real-Time PCR detection system (Bio-Rad) and a BIO-RAD iQ™ SYBR® Green Supermix Kit (Bio-Rad, Vienna, Austria). As a reference gene, glycerinaldehyde-3-phophate-dehydrogenase (*GAPDH*_fw: 5’aatcccatcaccatcttcca3’; *GAPDH*_rv: 5’cctgcttcaccaccttcttg3’) was used. For specific amplification of the respective transcripts, the following primer combinations were used: *KRT14*_fw: 5’TCCGCTGCGAGATGGAGCAG3’; *KRT14*_rv: 5’CATGACCTTGGTGCGGATTTGGC3’. *IL-1ß*_fw: 5’TTGAGAGGTGCTGATGTACCAGTTGG3’; *IL-1ß*_rv: 5’GTCCTGCGTGTTGAAAGATGATAAGCC3’. kallikrein-7: *KLK7*_fw: 5’TCAAGGCCTCGAAGTCATTC3’, *KLK-7*_rv: 5’GGTCAGAGGGAAAGGTCACA3‘; Rho guanine nucleotide exchange factor 1: *ARHGEF9*_fw: 5’AAGACCACAGTGACTACAGG3‘, *ARHGEF9*_rv: 5’TCCTCGCCCTCCCAGTCT3‘.

50 ng of cDNA and 15 pmol of each primer were added to each reaction. Reactions were prepared as master mixes and two reactions were analyzed per sample. Cycling conditions: 5 min at 95°C, 30 sec at 95°C, 20 sec at 60°C, 20 sec at 72°C, followed by melt curve analysis (8 sec / °C). Data were collected during each cycle and analyzed by the manufacturer’s software. Following the amplification, 5 μl of each RT-PCR product were analyzed on a 1 % agarose gel.

For the detection of the *trans*-splicing product in RV-RTM163-transduced EBDM-1 keratinocytes, a forward primer specific for the silent mutation in exon 7 (5’CAGGAGTACAAGATCCTGTTAGATGTCAAA3’) and an exon-8-specific reverse primer (5’CATCGTGCACATCCATGACCTTGGTG3’), cDNA of transduced cells, and GoTaq^®^ qPCR Master Mix (Promega, Mannheim, Germany) were used.

*Luciferase reporter assay*

A Dual-Luciferase® Reporter (DLR™) Assay System (Promega, Mannheim, Germany) was used to quantify *KRT14* promoter activity in the EBS-gen sev patient keratinocyte line EBDM-1 compared to RV_RTM163-expressing EBDM-1 cells. In the DLR Assay System, an experimental reporter enzyme, luciferase, from the firefly (*Photinus pyralis*) and a control enzyme (from *Renilla reniformis*) are expressed simultaneously in a single system. For generation of the experimental reporter, a full-length *KRT14* promoter (DQ343282) was cloned upstream of the firefly luciferase reporter gene of the pGL4.19 [luc2CP/Neo] vector (Promega). The *Renilla* luciferase on the control vector pRL-SV40 (Promega, Mannheim, Germany) is constitutively expressed under the control of an SV40 promoter. To quantify *KRT14* promoter activity, 1x10^6^ EBDM-1 or EBDM-1/RV_RTM163 cells were co-transfected with 1 µg experimental vector and 20 ng control vector by electroporation using Nucleofector™ Technology (Lonza, Basel, Switzerland), then seeded into 6-well plates and incubated at 37°C and 5% CO_2_. At 80-90% confluence, the cells were harvested and subjected to luminescence measurement according to the manufacturer’s protocol. The luminescence signal of the *Renilla* control vector served as an internal reference. In both cell lines the firefly luciferase signal was normalized to the *Renilla* signal (normSig = firefly/*Renilla*), and the fold change between the two cell lines was calculated based on normalized signals (normSigEBDM-1/RV_RTM163/normSigEBDM-1) according to the manufacturer’s normalization protocol.

*Total protein isolation*

Cells were washed three times with phosphate-buffered saline (PBS) (Dulbeccos’s PBS). 200 µl of cell-lysis buffer (0.5 M Tris-HCl, pH 6.8, 20% glycine, 10% SDS, 5% ß-mercaptoethanol; 1x complete protease inhibitor cocktail (Roche, Vienna, Austria)) were added. The lysate was passed through a 22G syringe to disrupt cellular aggregates and incubated at 95°C for 10 min. 1x Laemmli buffer (Bio-Rad, Vienna, Austria) was added.

*Immunoprecipitation and Western blotting*

For immunoprecipitation, Pierce Classic IP Kit (Thermo Fisher Scientific, Vienna, Austria) was used, according to the manufacturer’s protocol. As starting material, 10^6^ EBDM-1 or EBDM-1+RTM keratinocytes were used. For the preparation of the immune-complexes, 2 µL rabbit-α-GFP polyclonal IgG 598 from Medical and Biological Laboratories Co, Ltd (MBL, Woburn, USA) were used. For elution, 2 x non-reducing lane marker sample buffer with 5% ß-mercaptoethanol was used. Upon incubation at 95 °C for 10 min and centrifugation at 1,000 x g for 1 min.

20 µl cellular lysate or IP-eluate, respectively, were applied to a 4-12% NuPAGE Bis-Tris-Gel (Thermo Fisher Scientific, Vienna, Austria). Gels were run for 2 hours at 130 volts. Proteins were blotted onto Amersham Protran Premium 0.45 µm nitrocellulose membranes (GE Healthcare, Vienna, Austria) for 2 hours at 0.25 amperes. For blocking, Western Blocking Reagent (Roche, Vienna, Austria) was used. Primary antibodies: K14: mouse-derived monoclonal antibody LL001 (Santa-Cruz Biotechnologies); phospho-JNK: rabbit-anti-phospho-JNK (T183/Y185) (R&D Systems, Wiesbaden, Germany); α-actinin: rabbit-anti-α-actinin polyclonal IgG (H-300) (Santa-Cruz Biotechnologies, Heidelberg, Germany). Secondary antibodies: HRP-labeled rabbit-anti-goat, goat-anti-mouse and goat-anti-rabbit IgG2b antibody (DAKO, Frankfurt, Germany). HRP visualization was done using Amersham ECL Select Western Blotting Detection Reagent (GE Healthcare, Vienna, Austria) according to the manufacturer’s protocol.

*Generation of organotypic 3D skin equivalents*

Organotypic xenografts were prepared as previously described (S1). Briely, a fibrinogen matrix was produced by mixing 1x10^5^ wild-type human fibroblasts in 2.5 ml DMEM (Biochrom AG, Berlin, Germany) with 1.25 ml fibrinogen (25 mg/ml in 0.9% NaCl; Sigma, Vienna, Austria), 250 µl aprotinin (≡ 2.5 trypsin inhibitor units, TIU; Sigma, Vienna, Austria) and 250 µl thrombin (≡ 2.5 NIH units; Sigma, Vienna, Austria). The solution was mixed quickly and transferred to 6-well plates. The gel matrix was allowed to polymerize at 37°C, 5% CO_2_ in a humidified cell culture incubator. After 1 hour, 2.5x10^6^ keratinocytes of each test group were seeded onto the gel matrix in 2.5 ml of Green’s medium (S2). The skin equivalents were incubated at 37°C, 5% CO_2_ in a humidified environment for 3 to 4 days until the keratinocytes reached confluency.

*Immunofluorescence microscopy*

Skin sections derived from O.C.T biopsies were fixed in pre-cooled methanol for 10 minutes, washed with PBS and blocked with 5% bovine serum albumin (BSA) in PBS. Human type VII collagen was detected using a rabbit monoclonal antibody (kindly provided by Alexander Nyström, Freiburg), diluted 1:200 in 2% BSA/PBS overnight at 4°C. Second-step controls were not treated with the first antibody. As secondary antibody, an Alexa Fluor®488 goat-anti-rabbit IgG (H+L) antibody (Thermo Fisher Scientific, Vienna, Austria) was used at a 1:500 dilution. For nuclear staining, DAPI was used (VWR, Vienna, Austria), diluted 1:2000. Exposure time was optimized for the wild-type control and kept equal for all samples.

*Histo-morphological evaluation*

Grafts were harvested 8 weeks after transplantation. After short prefixation, skin explants were spanned on PBS-soaked gauze and four 4-mm punch biopsies were taken from each type of SE. The prefixative was freshly prepared phosphate-buffered 4% formaldehyde solution. Two punch biopsies were embedded in Tissue-Tek^®^ O.C.T. compound (VWR, Vienna, Austria) for immunofluorescence microscopy, and the other two were fixed in phosphate-buffered 4% formaldehyde solution for morphological and ultrastructural observation.

*Statistical analyses*

Statistical significance was evaluated by a one-way ANOVA including a Tukey’s multiple comparison test (sqRT PCR) as well as by a two-sided independent student’s *t*-test (promotor study) in GraphPad Prism.

**Supporting Material and Methods:**

*Morphological and ultrastructural observations by classical correlative light microscopy and transmission electron microscopy (CLEM)*

Skin specimens for light microscopical (LM) evaluation were analyzed by routine batch processing following strictly well-established procedures, comparable to those described in S3. Briefly, punch biopsies were fixed by a mixture of (phosphate-buffered) 2% formaldehyde / 2.5% glutaraldehyde in 0.13 M PO_4_-buffer, pH 7.4). Afterwards, orthogonally trimmed small skin tissue slices were chemically fixed in a phosphate-buffered 4% glutaraldehyde solution (pH 7.4). After washing, this was followed by secondary fixation/osmication (2% phosphate-buffered osmium tetroxide), washings and further processing by dehydration in an ascending ethanol gradient. Via acetonitrile (AN) as transition medium, the specimens were embedded in epoxy resin (glycidether 100, with components DDSA, MNA, and accelerator DMP-30; Serva, Germany) and thermally polymerized (S3-S5,). Semi-thin (1-μm thickness, LM) and ultrathin sections (approximately 70 nm to 80 nm in thickness) were cut using diamond knives (DIATOME Ltd, Biel, Switzerland). The semi-thin resin sections were stained with Azure II-Methylene Blue solution (AMbF) according to Humphrey and Pittman (S6), but modified for enhanced structure-discriminating polychromatic staining, and then photographed. Examination and documentation were accomplished using a ZETOPAN light microscope (Reichert, Vienna, vintage 1965).

*Fluorescence activated cell sorting (FACS):*

After transduction of EBDM-1 keratinocytes with the RV_RTM163_GFP, GFP positive cells were enriched by FACS, using the cell sorter FACS epics altsA (Beckman Coulter).

*DNA isolation and polymerase chain reaction*

Genomic DNA (gDNA) was isolated from punch biopsies by using the PureLink^TM^ Genomic DNA Mini Kit (Thermo Fisher Scientific, Vienna, Austria) according to the manufacturer’s protocol. 1 µg of gDNA was used as template to amplify the RTM using primers specifically annealing to the RTM’s silent mutations (RTMsilmut_fw: 5’ CAGGAGTACAAGATCCTGTTAGATGTCAAA 3’) and the BD (BD_rv: 5’ ACACTTACCCCTCCTCCAGTG 3’). PCR products were subjected to agarose gel electrophoresis, excised, purified (Illustra GFX PCR DNA and Gel Band Purification Kits, GE Healthcare) and sequenced. Sequencing was performed with an ABI Prism automated sequencer using an ABI PRISM dye terminator cycle sequencing kit (Thermo Fisher Scientific, Vienna, Austria).

*Immunofluorescence staining:*

For immunofluorescence staining to detect differentiation and proliferation skin sections derived from O.C.T biopsies were fixed in pre-cooled acetone for 5 minutes, washed with PBS and blocked with 2% bovine serum albumin (BSA) in PBS. Following primary antibodies were used and incubated for 1h at room temperature: anti K1 produced in rabbit (kindly provided by Prof. T. Magin), diluted 1:100 in PBS; anti K5 produced in rabbit (Covance), diluted 1:1000 in PBS; anti K14 produced in guinea pig (Progen), diluted 1:100 in PBS; anti Ki67 produced in rabbit (Thermo Scientific), diluted 1:100 in PBS. As secondary antibody Alexa Fluor®488 goat-anti-rabbit and Alexa Fluor®488 goat-anti-guinea pig antibodies (Thermo Fisher Scientific, Vienna, Austria) was used at a 1:400 dilution. For nuclear staining, DAPI was used (VWR, Vienna, Austria), diluted 1:2000.

**Supporting Information references:**

S1. Del Rio M, Larcher F, Serrano F, *et al*. A preclinical model for the analysis of genetically modified human skin in vivo. Hum Gene Ther 2002; **13**, 959-968.

S2. Rheinwald JG and Green H. Formation of a keratinizing epithelium in culture by a cloned cell line derived from a teratoma. *Cell* 1975*;* **6**, 317-330.

S3. Sadler E, Klausegger A, Muss W, *et al.* Novel KIND1 gene mutation in Kindler syndrome with severe gastrointestinal tract involvement. *Arch Dermatol* 2006; **142**, 1619-1624.

S4. Venable,J.H. and Coggeshall,R. (1965) A simplified lead citrate stain for use in electron microscopy. *J. Cell Biol.*, **25**, 407–408.

S5. Dingemans K.P. and van den Bergh Weerman M.A. (1990) Rapid contrasting of extracellular elements in thin sections. *Ultrastruct. Pathol.*, **14**, 519-527.

S6. Humphrey CD and Pittman FE. Methylene blue-azure II and basic fuchsin. *Stain Technol* 1974*;* **42**, 9-14.
